# Aww: The Emotion of Perceiving Cuteness

**DOI:** 10.3389/fpsyg.2016.01740

**Published:** 2016-11-10

**Authors:** Ralf C. Buckley

**Affiliations:** School of Environment, International Chair in Ecotourism Research, Griffith UniversityGold Coast, QLD, Australia

**Keywords:** cute, kawaii, cuteness, evolution, language

Emotions are one of the core components of human biology and behavior, linking senses, thoughts, and actions. In particular, emotions are one of the main components of human communications, including: facial expressions, postures and body language, pheromones and chemosignals, non-linguistic vocalizations, and spoken language (Lambie, 2009; Niedenthal and Brauer, 2012; Dehaene, 2014; Van den Stock and de Gelder, 2014; Lench et al., 2015; Ekman, 2016; Haviland-Jones et al., 2016; Martinez et al., 2016).

Neurophysiologists analyse human emotions as complex interlinked continua of neurological and hormonal signals and processes (Dehaene, 2014; Etkin et al., 2015; Bray, 2016). The social sciences, however, rely on the subtleties of language to differentiate and communicate the fine distinctions between closely related emotions (Johnson-Laird and Oatley, 1989; Ekman, 2016; Lakoff, 2016; Tissari, 2016). Some of these distinctions are very subtle, and we use languages to communicate those subtleties to each other.

For example, the emotion of fear is heavily studied (Buckley, 2016; Méndez-Bértolo et al., 2016), and there is a large family of English words related to the concept. These include: nouns describing specific emotions, such as anxiety, apprehension, trepidation, fright, terror, panic, and so on; adjectives describing either the source of fear or the person experiencing it, such as frightful or fearful; and adverbs and derived nouns, such as frighteningly and fearfulness.

As in many aspects of human communications, there are sometimes subtle cultural distinctions in the terminologies of communications. Kundera (1980), for example, argues that there is no exact English equivalent for the Czech word *litost*, a particular category of sadness. In general, however, there is a substantial degree of correspondence, between most modern languages, in the linguistic taxonomies of emotional constructs.

There is, however, one surprising omission. Across a number of major language groups, there are words for cute and cuteness, but no words for the human emotion experienced on perceiving cuteness. Terms such as *kawaii* in Japanese, or *mignon* in French, are sufficiently well-known to be adopted across cultures and languages. Indeed, the concept of *kawaii* includes an entire lexicon of associated subterms such as *mori, loli, kei*, and *decora*, which describe styles, fashions and personalities. There are terms for cute across languages with very different roots; *moni* in Spanish, *sudara* in Punjabi, *ke ai* in Chinese.

None of the languages listed above, however, contain a single term for the corresponding emotional response. Kawaii may translate as loveable, but love is not the emotion of cuteness, in the same way that happiness is not the same as awe (Coghlan et al., 2012; Darbor et al., 2015). Research in this field is forced to use portmanteau terms such as cute-emotion, cute-affect, or *kawaii*-feeling (Nittono et al., 2012; Laohakangvalvit et al., 2016; Nittono, 2016). In popular English, cute-emotion is represented by vocalizations such as *aww* or *d'aww*. *Aww* is listed in UK-English dictionaries, but only as an exclamation, not a noun.

Indeed, there is remarkably little published research on this emotion, relative to other human emotions. There are >1000 research publications on emotions such as fear (Buckley, 2016; Méndez-Bértolo et al., 2016), applying social, behavioral, physiological, and neurological as well as psychological perspectives. There are apparently <10, at least two orders of magnitude fewer, on cute-emotion (Nittono et al., 2012; Laohakangvalvit et al., 2016; Nittono, 2016).

Why should this matter? Language and terminology are powerful drivers of human attitudes and behaviors (Eskine, 2013), as demonstrated repeatedly in regard to racism (Yoo et al., 2009; Wei et al., 2015), gender (Formanowicz et al., 2015; Garnham et al., 2016; Hansen et al., 2016; Sczesny et al., 2016); crime (Hansen et al., 2016); and mental and physical health, body image and self-esteem (Yoo et al., 2009; Wei et al., 2015; Meadows and Daníelsdóttir, 2016). Names, or the lack of names, affect how people think and act.

This linguistic deficiency is particularly surprising, since cute-emotion has considerable biological significance. Cute-emotion is principally a response to neotenic or baby-animal characteristics, such as big round eyes, small size, and softness. These characteristics are involved in human mate selection (Jones and Hill, 1993; Jones et al., 1995; Perrett et al., 1998; Thayer and Dobson, 2013) and human parental care (Sherman et al., 2009; Nittono et al., 2012; Sprengelmeyer et al., 2013; Aragón et al., 2015; Golle et al., 2015; Hahn et al., 2016; though see also Sherman and Haidt, 2011).

Cute-emotion is a distinct and recognizable emotion, that complies with all the standard definitional criteria, such as characteristic hormonal responses, vocalizations, and facial expressions, recognizable across cultures and linked across senses (Röder et al., 2013). Its existence as an emotion, albeit as yet without a name, is recognized in psychological research (Lambie, 2009; Sherman et al., 2009; Winkielman et al., 2009; Sherman and Haidt, 2011; Nittono et al., 2012).

Cute-emotion is widely recognized and exploited in fashion, design (Huang et al., 2016; Noguchi and Tomoike, 2016), and marketing (Nittono et al., 2012; Nenkov and Scott, 2014; Nittono, 2016). In all these fields, commercial enterprises deliberately use cute characteristics to generate cute-emotion responses by their audiences or clientele. This is a powerful and effective approach. Similarly, cartoon movies and video games create cute characters to appeal to audiences and increase sales (Winkielman et al., 2009), and individuals preferentially watch videos of animals with cute characteristics (Nekaris et al., 2013).

Cute-emotion is thus recognized and used widely and pervasively in modern human societies. Despite this, it does not have a name, other than vernacular expressions such as “*aww”* in English. There are various possible reasons for this. The most plausible is that only modern civilizations have had the luxury of recognizing and responding to cuteness, through the deliberate design, manufacture, marketing and sale of items perceived as cute and used in social interactions. That is, even though the biological functions of cuteness in mate selection and parental care are longstanding, its social functions in peer esteem may be new.

With limited contrary evidence (Scott et al., 2014), biological responses to neotenic characteristics, as expressed in human mate choice and parental care, seem to be evolutionarily conservative, especially since similar characteristics are shared across many other species. The exaggerated representation of neotenic features, which contribute to cuteness in graphical and artistic representations, has precedents in ancient rock art (Achrati, 2014). It seems, therefore, that the biological functions of cute-emotion are not new.

The linguistics are more difficult to trace. The Japanese word *kawaii* is apparently >1000 years old, and the same probably applies for the Chinese term *ke ai*. It appears, however, that the precise meaning of the term has changed over time. In its earlier uses, it was applied in the literal sense to mean “loveable.” The much more specific associations of the modern usage of *kawaii*, e.g., in dress, fashion and toys, has only evolved recently. The widespread change in clothing styles from traditional *kimono* to the plethora of Japanese dress fashions such as *mori, loli*, and *kei* has taken place within the last half-century. The dissemination and stylistic cross-fertilization between Japanese *kawaii* fashions and other cultures is equally recent. Adoption of *kawaii* styles in toys and design has also expanded largely within the last few decades. During this same period of rapid and recent cultural linguistic evolution, the meaning of the term “cute” in English has also become diversified. The term can be used in a pejorative as well as an appreciative sense, and this may have blocked the evolution of a term for cute-emotion.

The use of cute-emotion in social interactions, however, is now widespread and pervasive in Western as well as Eastern cultures and countries. As just one example not mentioned above, marketing materials for wildlife tourism products focus heavily on images of young animals which generate cute-emotion in potential purchasers (Buckley and Mossaz, 2016). Given the significance of cute-emotion in biology, society, and commerce, I propose that it deserves substantially greater attention in psychological research, building on existing studies cited here, and adopting all the methodological approaches used widely in studying other human emotions. I suggest that one possible reason for the limited attention it has received to date, relative to its social significance, is that it has not had a simple and widely recognized formal name. Attitudes and behavior, for research psychologists as for everyone else, are influenced by language. To overcome this obstacle, I propose that the most common English vernacular term, *aww*, should be adopted as a formal name for cute-emotion.

## Author contributions

The author confirms being the sole contributor of this work and approved it for publication.

### Conflict of interest statement

The author declares that the research was conducted in the absence of any commercial or financial relationships that could be construed as a potential conflict of interest.
